# R-Based Software for the Integration of Pathway Data into Bioinformatic Algorithms

**DOI:** 10.3390/biology3010085

**Published:** 2014-02-07

**Authors:** Frank Kramer, Michaela Bayerlová, Tim Beißbarth

**Affiliations:** University Medical Center Göttingen, Department of Medical Statistics, Humboldtallee 32, D-37073 Göttingen, Germany; E-Mails: michaela.bayerlova@med.uni-goettingen.de (M.B.); tim.beissbarth@med.uni-goettingen.de (T.B.)

**Keywords:** Pathway data, data integration, R-project, bioconductor, BioPAX, rBiopaxParser, Cytoscape

## Abstract

Putting new findings into the context of available literature knowledge is one approach to deal with the surge of high-throughput data results. Furthermore, prior knowledge can increase the performance and stability of bioinformatic algorithms, for example, methods for network reconstruction. In this review, we examine software packages for the statistical computing framework R, which enable the integration of pathway data for further bioinformatic analyses. Different approaches to integrate and visualize pathway data are identified and packages are stratified concerning their features according to a number of different aspects: data import strategies, the extent of available data, dependencies on external tools, integration with further analysis steps and visualization options are considered. A total of 12 packages integrating pathway data are reviewed in this manuscript. These are supplemented by five R-specific packages for visualization and six connector packages, which provide access to external tools.

## 1. Introduction

Easier access and decreased costs have lowered the entrance barrier for performing high-throughput experiments. However, analysis and interpretation of this data poses a challenge for biologists and bioinformaticians alike. This surge in generation of new data, both *in vitro* and *in vivo*, will naturally entail a surge in newly generated results as well. Such results can be used in the discovery of new drug targets, protein-protein interactions or regulatory effects. In either case, putting new findings into context of already existing knowledge is essential. An important aspect to evaluate results of high-throughput experiments is access to pathway data within the scope of programming environments. There are several methods incorporating pathway data into these analyses either in a form of the gene sets [1,2] or as regulatory graphs [3,4,5]. Furthermore, access to pathway data enables researchers to programmatically verify their results, for example by putting new results into context of available literature knowledge and thus testing for overlaps of findings with prior knowledge [6]. Additionally, the integration of pathway knowledge is also increasingly important for methods and approaches in bioinformatics. Many bioinformatic algorithms can increase their power and robustness, if prior knowledge is directly integrated during the analysis [7,8,9].

This manuscript aims to give an overview of software packages for the R Project for Statistical Computing that integrate pathway data for bioinformatic analysis within R. This manuscript focuses on provided pathway data, the extent of this data, and the integration of the R package with further analysis steps, as well as procedures for data visualization.

### 1.1. Modeling Pathway Knowledge

In general, pathways can be divided into the groups of metabolic pathways, signaling pathways, gene regulatory networks and protein–protein interaction networks, however, mixtures and overlaps exist. Metabolic pathways represent chains of chemical reactions of small molecules, so called metabolites, which are catalyzed by specific proteins, called enzymes. Signaling pathways represent the communication within and between cells. Gene expression is the process of generating the functional cell product of the DNA sequence of a gene. Gene regulatory networks model the indirect regulation and interaction of genes. Further, protein–protein interaction (PPI) databases store computationally inferred or experimentally validated protein–protein interactions, which can be assembled to PPI networks. Certain design decisions have to be made to model any of the pathway types, mainly coming down to a trade-off between richness of detail and generalization. Several ways to encode pathway knowledge have been developed to represent the various pathway types. Apart from proprietary XML or database models developed by pathway providers, a number of standards have received broader attention. The KEGG Markup Language (KGML), developed for and used by the Kyoto Encyclopedia of Genes and Genomes (KEGG) database, is an example for a proprietary data model of a database provider to encode metabolic and signaling pathways [10]. However, generic and well-documented formats, which aim at easing knowledge exchange, have been proposed in recent years. The Systems Biology Markup Language (SBML) [11] is designed to represent quantitative pathway models, as well as metabolic and regulatory pathways. Its main focus is on encoding quantifiable models to be used in systems biology modeling. The ontology for Biological Pathway Exchange (BioPAX) [12,13] models pathway knowledge and aims at promoting knowledge exchange between researchers. The latest release BioPAX Level 3 is able to represent metabolic, signaling as well as regulatory pathways. The Human Proteome Organization (HUPO) published the Proteomics Standards Initiatives Molecular Interaction (PSI MI) format, which initially only encoded protein–protein interaction data [14]. However, the format has been extended and can model various chemical entities and molecular interactions and features a querying syntax [15,16].

### 1.2. Pathway Databases

A plethora of pathway databases are freely available on the web. Pathguide [17], an online resource listing pathway database, currently contains links to over 300 different databases. One of the most notable databases, featuring metabolic and signaling pathways, is the Kyoto Encyclopedia of Genes and Genomes (KEGG) [10]. However, access to the bulk FTP download of data was restricted in 2011 and is currently only available via a subscription fee (http://www.kegg.jp/kegg/docs/plea.html). On the other hand, pathway sketches and data can still be retrieved free of charge via the KEGG website and web services. Reactome [18] is an open-source pathway database, manually curated and peer-reviewed, which is available in many different data formats, for example SBML, BioPAX and as MySQL-dump. The Pathway Interaction Database (PID) [19] is a collaborative project of the National Cancer Institute (NCI) and the Nature Publishing Group launched in 2006. As of 22 September, 2012 the NCI has taken over the project and will perform future updates. PID is available encoded in proprietary XML and in BioPAX Level 2 and Level 3. WikiPathways [20] on the other hand, is a community approach to pathway curation, available in BioPAX and proprietary formats. For a detailed review see Bauer-Mehren and colleagues [21], who evaluated popular databases concerning their extent and overlaps for specific pathways, including KEGG, Reactome, WikiPathways and PID. The European Bioinformatics Institute (EBI) hosts a registry (http://www.ebi.ac.uk/Tools/webservices/psicquic/registry/registry?action=STATUS) currently listing 28 web services available for querying databases for PSI MI-compatible data using the PSICQUIC querying language [16].

### 1.3. Tools for Pathway Curation and Analysis

During the last decade, a number of tools have been developed to create, edit and analyze pathways. Most notable among these are CellDesigner, PathVisio and Cytoscape. CellDesigner is heavily influenced by the Systems Biology Graphical Notation (SBGN) [22] and focuses on pathway diagram creation. PathVisio [23] and Cytoscape [24] are Java-based open-source tools to curate and analyze pathways. Both can be extended by a number of plugins to support more data formats or integrate new pathway analyses. Furthermore, both PathVisio and Cytoscape feature functionality to execute remote procedure calls and exchange and visualize data via external programs. The corresponding R package *RCytoscape* [25] is part of this review for its data loading and visualization functionality. Comparable PathVisio code can be found in an online tutorial (http://projects.bigcat.unimaas.nl/pathvisiorpc/tutorial). Arguably, Cytoscape is currently the most popular tool for pathway editing, featuring over 150 plugins in the Cytoscape App store [26]. An extensive review of standalone tools for pathway curation has been compiled by Sunderman and Hallett [27].

### 1.4. R Framework for Statistical Computing

The R Framework for Statistical Computing [28] has been well established in the field of bioinformatics and features a variety of tools to perform pathway analysis [8,9], methods for network reconstruction [29,30], as well as libraries for visualizing graphs and biological networks [31]. The main resources for R packages are the online repositories Comprehensive R Archive Network (CRAN) [32], Bioconductor [33] and the Omega Project for Statistical Computing [34], which currently contain 4705, 671 and 98 packages respectively. Categorized lists of packages for certain tasks, as well as search functionality allow the user to browse these repositories easily. However, it is hard to judge which package is the right one for a given task just by the package name and short description.

## 2. Methods Section

Within this review R packages are evaluated according to five different aspects: The first aspect determines the source of integrated data; renowned metabolic or signaling pathway databases like KEGG or Reactome are more often integrated and available via several packages. Within the second aspect, the internal data model and its extent are described; this can range from supplying only gene sets of pathways, to undirected graphs and directed graphs with fully annotated edges. The next criterion will assert the dependence or interaction of the R package with external tools for tasks such as visualization or data import; for example Cytoscape [24] or the Graphviz [35] libraries. The fourth aspect describes whether further methods for analyses, e.g., pathway analyses or methods for network reconstruction are already included or can be easily integrated by supplying readily transformed pathway data as input. Finally, visualization strategies will be described, ranging from no extra functionality to complex and colorful plots by using internal R functionality, external tools such as Cytoscape, or other graphical user interfaces.

### 2.1. Overview of Available Packages

The two most notable online repositories for R packages, CRAN [32] and Bioconductor [33], have been screened for packages which integrate pathway data. Various approaches to integrate, process and visualize pathway data have been realized by the package authors. A total of 12 packages integrating pathway data have been identified and are described here. These are supplemented by 5 R-specific packages for visualization, for example *Rgraphviz*, and 6 connector packages, for example *XML* and *RJava*, which provide access to external tools. Table 1 offers a list of reviewed packages along with their main features and properties.

**Figure 1:**
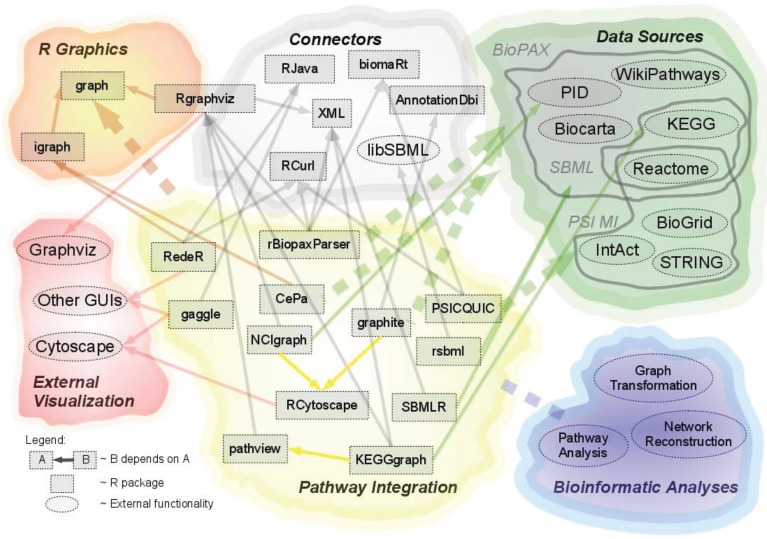
This figure illustrates the dependencies and interactions of R packages, pathway data sources, as well as packages acting as connectors between the different modules.

In order to generate an overview of popular tools promoting the use of pathway data in R, the dependencies of and between these packages have been depicted in a dependency network in Figure 1, see Section 2.4. “Dependency on external tools”.

**Table 1:** This table lists the reviewed packages for integrating pathway data into R. Packages and are stratified according to the aspects of data sources, strategies of data import, dependencies on external tools, integration with further bioinformatic analyses and visualization opportunities.

Package Name	Data Source	Data Import	Dependencies	Further Analyses	Visualization
**rBiopaxParser**	generic BioPAX parser; all BioPAX databases	gene sets, directed graphs, full annotation	XML, biomaRt		Rgraphviz
**graphite**	includes KEGG, BioCarta, PID, Reactome, SPIKE	gene sets, directed graphs, mapping and converting IDs	AnnotationDbi	Pathway analyses: clipper, SPIA	Cytoscape
**NCIgraph**	load PID data via Cytoscape	graph objects with directed edges	Java, Cytoscape		Rgraphviz
**pathview**	load data via KEGGgraph	gene sets with graph layout annotation	KEGGgraph	Pathway analyses: gage	Rgraphviz + native KEGG
**KEGGgraph**	generic KGML parser, KEGG	graph objects with directed edges	XML, biomaRt		Rgraphviz
**RedeR**		igraph objects	Java		Java GUI
**SBMLR**	generic SBML parser, limited functionality	list of SBML class instances	XML	deSolve	-
**rsbml**	generic SBML parser	graph objects	libSBML	SBML ODE Solver Library (SOSLib)	Rgraphviz
**RCytoscape**	load data via Cytoscape, R	graphNEL objects	Java, Cytoscape		Cytoscape
**Gaggle**	load data via Gaggle server	graph objects with directed edges	Gaggle		-
**CePa**	includes KEGG, BioCarta, PID, Reactome	igraph objects	igraph	Pathway analyses.GSEA, ORA	igraph
**PSICQUIC**	PSI MI-QL compliant databases	list of interactions	RCurl		

### 2.2. Source of Integrated Pathway Data

The common sources of data for all reviewed R packages are online pathway databases, which allow users to download an export of their curated pathway data or access via a web service. All reviewed packages pursue one of three approaches to integrate pathway data into R: The first approach is that pathway data is retrieved via external tools, i.e., Gaggle [36] or Cytoscape [24], which allow the user to access data types compatible with these tools. For example the package *NCIgraph*, tailored around the Pathway Interaction Database (PID) [19] of the National Cancer Institute (NCI), bases on *RCytoscape* to load pathways via Cytoscape to import arbitrary BioPAX data. The *RCytoscape* package [25] is able to retrieve networks within a Cytoscape window as R graph objects using generic remote procedure calls via the *XMLRPC* package. Furthermore, R graph objects can be loaded and visualized via Cytoscape. Another example is the R package *gaggle*, which is able to link to a Gaggle host and receive networks from other applications using the Gaggle framework for data exchange.

The second approach is that pathway data is retrieved, parsed and curated in a more or less automatic manner by the package maintainers and subsequently distributed directly via the package or as separate download. This approach is applied by the *graphite* package [37], which includes the pathway data of KEGG [10], BioCarta [38], PID [19], Reactome [18] and SPIKE [39]. The same approach is applied by the *CePa* package [40], which includes KEGG, BioCarta, PID and Reactome. Package *pathRender* reads interaction lists from the *cMAP* data package and builds graphs from these interactions.

Finally, the third approach is the ability of packages to parse data encoded in generic pathway formats and model these in an internal R representation. Naturally, this enables users to parse arbitrary pathway databases. The *KEGGgraph* package parses KGML encoded data and supplies it as R objects package [41]. The *Pathview* package [42] loads KGML encoded files using the *KEGGgraph* package and also features a download function, which is able to automatically retrieve KEGG pathways via the web. The *SBMLR* package [43] parses SBML up to Level 2 using the R *XML* package. The package *rsbml* uses the system library libSBML [44] to parse, validate and convert SBML data to R graphs, objects and currently supports SBML up to Level 3. The package *PSICQUIC* [45] retrieves interactions and annotations in the PSI MI format via web services offering PSI MI query capabilities and returns these as lists of interactions within R. Finally, the *rBiopaxParser* parses arbitrary BioPAX Level 2 and Level 3 databases using the *XML* package and represents them in R [46].

### 2.3. Internal Data Model

Another criterion for pathway data integration is the extent of data that is made available by individual packages. The simplest representation of a pathway in R is a gene set. Further, undirected or directed graphs can be used to represent a specific pathway. In order to integrate the pathway data into further analyses, the user has to ensure that the appropriate information about nodes, edges and edge direction is available. Depending on the package, pathway graphs might be available in a mechanistic fashion, including all biological processes, e.g., complex assembly, cellular transport, transcription or phosphorylation, or pathways might be transformed into a regulatory graph, only containing inhibiting or activating interactions. Furthermore, the data provided by the databases might be enriched by additional annotation data for genes or references to published evidence.

The *pathview* and *KEGGgraph* packages supply for every pathway a KEGGPathway object, which includes pathway information like title and organism, and a directed R graph object, which maintains the pathway topology and all essential attributes. Both metabolic and regulatory graph views can be extracted from the KEGGPathway object. Furthermore, pathway nodes in KGML can represent families of proteins, which the packages are able to expand and map to the corresponding set of single proteins. Cytoscape is a Java-based software platform for visualizing and analyzing networks and graphs, which can be extended by a large number of available plugins. *RCytoscape* retrieves networks from Cytoscape and represents them as R graph objects. The R object has directed or undirected edges and is annotated depending on the network within Cytoscape. The Gaggle framework consists of plugins for different software tools and enables dynamic data exchange via a host service, which connects the independent tools. Package *gaggle* can receive network data from other Gaggle applications and import these as graph objects. *NCIgraph* bases on the data retrieved via *RCytoscape* and offers further functionality to merge pathway nodes of PID data, which depends on a specific annotation by the NCI. Despite the similar naming, the packages providing SBML data, *rsbml* and *SBMLR*, use different approaches for their internal data model. *SBMLR* parses the pathway data into an internal model comprised of a set of lists. These are specifically tailored to represent reactants, products and the reaction rates for model simulations in systems biology. On the other hand, *rsbml* uses libSBML to parse an SBML model either as an R graph object or as a Document Object Model (DOM). Using convenience functions the annotation data within the DOM object can be queried and modified, the graph object can be extracted and used for further analyses. The *graphite* package offers its parsed data as pathway objects, which are imported from KGML- and BioPAX-encoded data and converted to R objects. These objects store information about the nodes and directed and undirected edges, as well as the data source, pathway name and species. The pathway objects can be converted into R graph objects. Similarly, the *CePa* package supplies pre-parsed interaction lists of its integrated databases and can generate directed igraph pathway graphs. The *PSICQUIC* package enables the users to retrieve PSI MI-compatible lists of molecular interactions from databases offering PSICQUIC web services. Afterwards these can be used to assemble networks or graphs from the retrieved interactions and annotations. The package *rBiopaxParser* parses arbitrary BioPAX-encoded data and provides biopax objects within R. These objects include all information of the original data within one data frame, similar to the triplets of the Resource Description Framework (RDF) model. The BioPAX data can be accessed and edited using convenience functions and pathways can be converted into R graph objects.

### 2.4. Dependency on External Tools

A number of packages for pathway integration rely on external tools, either for accessing, parsing and importing data or for mapping between various identifiers. Many packages depend on external tools for visualization purposes as well; however, these are described in Section 2.6. “Visualization of Pathway Data”. Figure 1 illustrates the dependencies of and between the data sources, reviewed packages, as well as the external tools.

Not all dependencies must be fulfilled to run the packages out of the box, however functionality might be crippled. In order to use the download functionality of the *rBiopaxParser* package and the querying capabilities of *PSICQUIC*, the curl tool must be installed. The *gaggle* package for data exchange relies on Java and the *rJava* package for communication with other Gaggle applications. The R packages *rBiopaxParser*, *KEGGgraph* and *SBMLR* rely on the *XML* package and the libxml system library for parsing, similarly *rsbml* depends on libSBML. For data import the packages *NCIgraph* and *RCytoscape* depend on a running Cytoscape session to load networks. In order to enrich available annotation and map identifiers the packages *graphite* and *pathview* depend on the *AnnotationDbi* package, while *KEGGgraph*, *PSICQUIC* and *rBiopaxParser* depend on the *biomaRt* package and web service.

### 2.5. Integration with Further Analysis Steps

Pathway analyses in its classical approach, the gene set enrichment analysis, allow bioinformaticians to test if a pathway is significantly regulated between groups of samples. Many different algorithms are available in R to perform gene set enrichment analyses, for example via package *HTSanalyzeR* [47] and CAMERA in the *limma* package [48,49]. More advanced pathway analysis tools are able to take pathway topology into account, e.g., SPIA [3], clipper [4], GGEA [50] and many other available as R packages from Bioconductor [5,51,52,53]. Furthermore, methods for network reconstruction aim at reconstructing networks by analyzing gene co-expression or intervention data [54]. Different approaches have been published and were implemented as R packages. Several of these can be enhanced in their performance by integrating graphs as prior knowledge, for example *NEM* [7], *DDEPN* [30], *birta* [55] and *BoolNet* [56]. On the other hand, packages offering access to SBML-encoded data, which is focused on describing quantitative models and enable direct integration with solvers for ordinary differential equations (ODEs) [57,58].

Almost all reviewed packages enable the user to access pathways within R as graph objects. If nodes in these graphs represent genes, a list of nodes can be used to perform gene set enrichment analyses. For more advanced algorithms the input has often to be reformatted to match the requirements of the specific implementation. Graphs might have to be either directed or undirected, edge weights must stay within certain limits and different edge types might or might not exist. In order to enhance the bioinformatic workflow several reviewed packages offer a kind of wrapper for further analyses. Graphs are automatically transformed into the right format and the analyses can be directly called from within the package. The packages *rsbml* and *SBMLR* include wrappers for solving ODEs using the SBML ODE Solver Library SOSlib [57] and the R package *deSolve* [58] respectively. The *graphite* package enables users to run several pathway analyses tools which take pathway topology into account for their testing procedures, for example clipper [4], SPIA [3] and DEGraph [59]. The *CePa* package integrates standard gene set enrichment and custom over-representation analyses published by Gu and colleagues [40].

### 2.6. Visualization of Pathway Data

There are several packages available which focus primarily on the visualization of R graph data. Package *pathRender* provides plotting capabilities with preset parameters to quickly retrieve visualization of graphs. *RedeR* [60] is a powerful tool, which implements plotting and remote interaction of graph objects of package *igraph* with a Java-based graphical user interface similar to Cytoscape, especially focused on visualizing extremely large networks. *CePa* provides preset plotting parameters for its supplied igraph objects. The packages *NCIgraph*, *KEGGgraph*, *pathview* and *rBiopaxParser* enable the user to plot pathways via *Rgraphviz*. Package *pathview* additionally provides a function to export native KEGG representations as PNG image files. The *graphite* package, which uses *RCytoscape* for visualization, as well as *RCytoscape* itself, visualize graphs via an active Cytoscape session. *RCytoscape* offers a multitude of well-documented parameters and functions to visualize, layout and modify R graph objects using Cytoscape.

Furthermore, the user is free to extend and improve visualization, since all methods for visualization—Cytoscape, RedeR, Graphviz as well as common R plotting—allow for individual modifications of parameters, e.g., color, size of nodes, width of edges.

## 3. Summary

Bioinformatics and systems biology are still rapidly evolving fields, and along with these many new and exciting software tools are becoming available. This speed as well as the interdisciplinary work makes it difficult for biologists and bioinformaticians alike to keep up with new developments. A positive aspect is the maturing of data sources for pathway knowledge: All mentioned database providers either offer exports abiding to the standards of SBML [11] or BioPAX [12], or even use the standards internally.

However, the internal data model of the reviewed packages is usually a combined use of the R graph class and additional, package-specific, tables or lists for further annotation data, for example for identifier mapping or edge and node types. None of the dependencies on external tools really restrict the use of the reviewed packages. The standalone versions of Cytoscape, RedeR and Gaggle are available via Java on all platforms and the Graphviz library has been integrated into the *Rgraphviz* package since version 2.1. This enables many differently flavored visualization options from within R.

Assessing pathway analyses or network reconstructions probably pose the biggest challenge for users, due to the package-specific data modeling of pathways within R. This is partly due to the fact that the packages for further analyses have varying requirements on graphs or prior knowledge, for example, that graphs must be supplied as undirected graphs or directed acyclic graphs. However, these problems might be tackled in the future by providing standardized pathway classes, for example via Bioconductor, which can automate these transformations.

Concerning the visualization, it is not surprising that packages which mostly act as an interface and connect R and external tools such as *RCytoscape* and *RedeR* are mainly focused on providing general capabilities for data exchange and visualization to their users. On the other hand, packages which focus on providing pathway information, for example *graphite*, *CePa*, *rBiopaxParser* or *KEGGgraph*, provide plotting capabilities with many preset parameters and thus offer pre-formatted pathway visualization. This enables users to work with these packages and the provided pathway data out of the box.

All of the reviewed R packages have a clear focus and provide unique capabilities. However, the different aspects within this review should enable users to narrow down their choice of packages for pathway data within R, according to the desired data sources, pathway analyses options and possibilities for visualization.

## 4. Conclusions

Although, this review is focused on pathway data within R, users may also choose to fully rely on other tools. The Cytoscape platform has a large community of users and numerous plugins in its new app store [26]. These enable users to import and curate pathway data, conduct analyses and offer many visualization possibilities. On the other hand, web tools such as DAVID [61] or Graphite Web [62] aim at providing a maximum of convenience by offering simple access to basic and advanced high-throughput analyses, as well as visualization via the web browser.

Programming in R leaves many options for interactions with different systems and services and near endless possibilities to perform and combine analyses and tools. However, compared to all-in-one tools like DAVID, these possibilities come at a price: Even with the emerging generic standards for data encoding like BioPAX, SBML or PSI MI, the availability of interfacing or libraries for various programming languages can be a limiting factor. Fortunately, new interfaces [16] and libraries [46] will appear over time and gaps between data formats will become smaller [15,63]. Strömbäck and Lambrix [64] compared different encodings of pathway knowledge, evaluating the features and capabilities of SBML, PSI MI and BioPAX. Cary and colleagues reviewed available pathway information and different data formats for encoding pathway knowledge [13]. Furthermore, a number of extensions and approaches to convert between formats have been published [65,66,67,68,69]. Unfortunately, lossless knowledge conversion between standards and reproducibility of pathway curation/reconstruction efforts remains hard to achieve.
